# Phosphoproteomics of Retinoblastoma: A Pilot Study Identifies Aberrant Kinases

**DOI:** 10.3390/molecules23061454

**Published:** 2018-06-15

**Authors:** Lakshmi Dhevi Nagarajha Selvan, Ravikanth Danda, Anil K. Madugundu, Vinuth N. Puttamallesh, Gajanan J. Sathe, Uma Maheswari Krishnan, Vikas Khetan, Pukhraj Rishi, Thottethodi Subrahmanya Keshava Prasad, Akhilesh Pandey, Subramanian Krishnakumar, Harsha Gowda, Sailaja V. Elchuri

**Affiliations:** 1 L&T Opthalmic Pathology, Vision Research Foundation, Sankara Nethralaya, Chennai, Tamil Nadu 600 006, India; lakshmi.nagarajhan@gmail.com (L.D.N.S.); ravikanthdanda@gmail.com (R.D.); drkk@snmail.org (S.K.); 2 Centre for Nanotechnology and Advanced Biomaterials, Shanmugha Arts, Science, Technology and Research Academy University, Tanjore, Tamil Nadu 613 401, India; umakrishnan@sastra.edu; 3 Institute of Bioinformatics, International Technology Park, Bangalore, Karnataka 560 066, India; anil@ibioinformatics.org (A.K.M.); vinuth@ibioinformatics.org (V.N.P.); gajanan@ibioinformatics.org (G.J.S.); keshav@ibioinformatics.org (T.S.K.P.); pandey@jhmi.edu (A.P.); 4 Manipal Academy of Higher Education (MAHE), Manipal, Karnataka 576 104, India; 5 Shri Bhagwan Mahavir Vitreoretinal Services, Sankara Nethralaya, Chennai, Tamil Nadu 600 006, India; drvk@snmail.org (V.K.); drpr@snmail.org (P.R.); 6 Center for Systems Biology and Molecular Medicine, Yenepoya Research Centre, Yenepoya (Deemed to be University), Mangalore, Karnataka 575 108, India; 7 McKusick-Nathans Institute of Genetic Medicine, Johns Hopkins University School of Medicine, Baltimore, MD 21205, USA; 8 Departments of Biological Chemistry, Pathology and Oncology, Johns Hopkins University School of Medicine, Baltimore, MD 21205, USA; 9 Department of Nanotechnology, Vision Research Foundation, Sankara Nethralaya, Chennai, Tamil Nadu 600 006, India

**Keywords:** ocular cancer, DEK, oncogenic kinases, DNA damage response, H2AFX

## Abstract

Retinoblastoma is a malignant tumour of the retina which most often occurs in children. Earlier studies on retinoblastoma have concentrated on the identification of key players in the disease and have not provided information on activated/inhibited signalling pathways. The dysregulation of protein phosphorylation in cancer provides clues about the affected signalling cascades in cancer. Phosphoproteomics is an ideal tool for the study of phosphorylation changes in proteins. Hence, global phosphoproteomics of retinoblastoma (RB) was carried out to identify signalling events associated with this cancer. Over 350 proteins showed differential phosphorylation in RB compared to control retina. Our study identified stress response proteins to be hyperphosphorylated in RB which included H2A histone family member X (H2AFX) and sirtuin 1. In particular, Ser140 of H2AFX also known as gamma-H2AX was found to be hyperphosphorylated in retinoblastoma, which indicated the activation of DNA damage response pathways. We also observed the activation of anti-apoptosis in retinoblastoma compared to control. These observations showed the activation of survival pathways in retinoblastoma. The identification of hyperphosphorylated protein kinases including Bromodomain containing 4 (BRD4), Lysine deficient protein kinase 1 (WNK1), and Cyclin-dependent kinase 1 (CDK1) in RB opens new avenues for the treatment of RB. These kinases can be considered as probable therapeutic targets for RB, as small-molecule inhibitors for some of these kinases are already in clinical trials for the treatment other cancers.

## 1. Introduction

Phosphorylation is one of the major post-translational modifications of proteins that regulates many cellular processes, including cellular communication, proliferation, differentiation, and survival. This is emphasized by the fact that about one third of all eukaryotic proteins are phosphorylated at some point [1]. Protein phosphorylation is governed by the coordinated function of kinases and phosphatases. Dysregulated kinase signalling is observed in many diseases, including cancer [2]. Kinases are the key phosphoproteins, as they are central in various cellular signalling processes. There are 518 kinases identified in the human genome [3], and studying the kinome map under diseased conditions has become a research focus in the last decade. 

Several methods are used for studying phosphoproteomics and deducing signalling networks, such as antibody-based arrays [4], mass spectrometry [5,6], and flow mass-cytometry [7]. Due to the limited availability of antibodies for phosphoproteins, mass spectrometry has emerged as the most prominent technology for analysing kinome-regulated signalling networks [8,9]. Phosphoproteomics is an emerging tool in the study of cancer for the identification of potential biomarkers and potential therapy targets [10,11,12]. Phosphoproteomic approaches have identified targets for early screening and therapy in hepatitis C virus (HCV)-mediated hepatocellular carcinoma [13]. Phosphoproteomics has been applied to understanding disease progression and therapy in breast, lung, and brain cancers [14,15,16].

Retinoblastoma (RB) is a childhood retinal cancer, with an incident rate of about 1 in 15,000–18,000 live births. RB occurs due to the lack of a functional *RB1* gene, usually resulting in enucleation in developing countries due to the diagnosis of the disease at later stages. Studying the disease progression of RB using omics approaches and identifying novel therapeutic targets has recently been a primary research focus. Genomic, genetic, and epigenetic changes have been studied widely to characterize RB tumours in studies which concentrated on mutations, differential gene expression, methylation changes, and deregulated miRNAs in RB [17]. These studies evaluated differentially expressed genes in RB, leading to the identification of candidate oncogenes and tumour suppressors involved in the progression of retinoblastoma. Some of these genes were also proposed as potential therapeutic and prognostic targets. However, these studies did not focus on the signalling pathways activated in RB. Protein phosphorylation is a critical determinant of signal transduction pathways. We carried out a pilot quantitative phosphoproteomics study of primary RB tumour tissues and control retina to identify crucial proteins in the signal transduction pathways of RB. This study identifies the phosphorylated proteins in human retina for the first time in addition to specific sites that are regulated in retinoblastoma. This study should help as an initiation factor for subsequent studies to investigate and identify molecular mechanisms governing RB progression and identify novel biomarkers and therapy options for retinoblastoma. 

## 2. Results

### 2.1. Phosphoproteome of Human Retina

The phosphoproteome of the human retina has not been studied thus far. Phosphoproteomic analysis of retinoblastoma and retina identified 1393 proteins that were phosphorylated in both tissues. These phosphoproteins (irrespective of their differential expression status) were represented by 2568 unique phosphopeptides containing 3476 phosphosites. The frequency of the identified phosphorylated sites was Ser (91%), Thr (8%), and Tyr (1%). 

**Figure 1:**
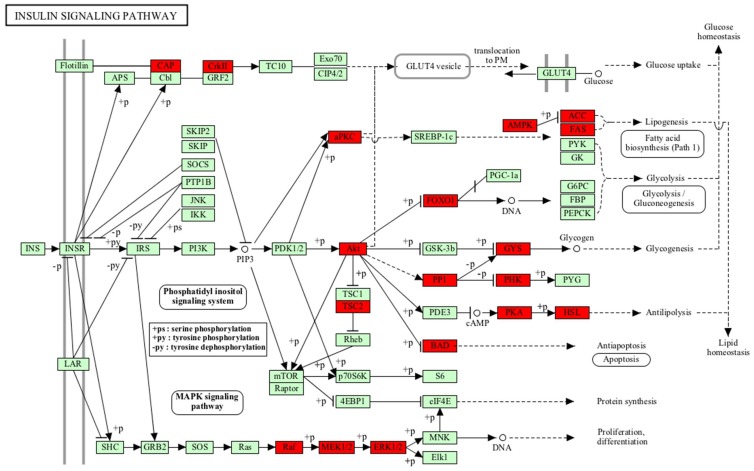
Insulin signalling pathway activated in retina and RB. Akt mediated downstream signalling of FOXO1 and BCL-2 antagonist of cell death (BAD) play different roles in retina and retinoblastoma. The insulin pathway has a cell survival role rather than acting as a storage pathway in retina and retinoblastoma.

To obtain insight into signalling events occurring in retina, phosphorylated proteins identified in both retina and retinoblastoma were analysed by the DAVID tool (https://david.ncifcrf.gov/). The phosphoproteome revealed an enrichment of pathways involved in spliceosome, tight junction, and insulin signalling (Table 1). Important mediators of the insulin pathway such as Akt, BCL-2 antagonist of cell death (BAD), FOXO1, and FASN were found to be phosphorylated in both retina and retinoblastoma tissues (Figure 1). Tight junction proteins, which play a major role in the formation of the blood retinal barrier, were also found to be phosphorylated. 

### 2.2. Differentially Phosphorylated Proteins in Retinoblastoma

After identifying the signalling pathways present in both retina and retinoblastoma, we focused our analysis on phosphoproteins that are differentially phosphorylated in RB compared to retina. The analysis identified about 508 phosphosites corresponding to 361 unique proteins that were differentially phosphorylated. Phosphorylated peptides with a fold change of 1.5 in at least three tumour samples were considered to be differentially phosphorylated. We found 158 proteins to be hyperphosphorylated and 203 proteins to be hypophosphorylated in retinoblastoma compared to control retina (Supplementary Tables S2 and S3). 

### 2.3. Categorization of Differentially Phosphorylated Proteins Based on Gene Ontology (GO) Annotation

**Figure 2:**
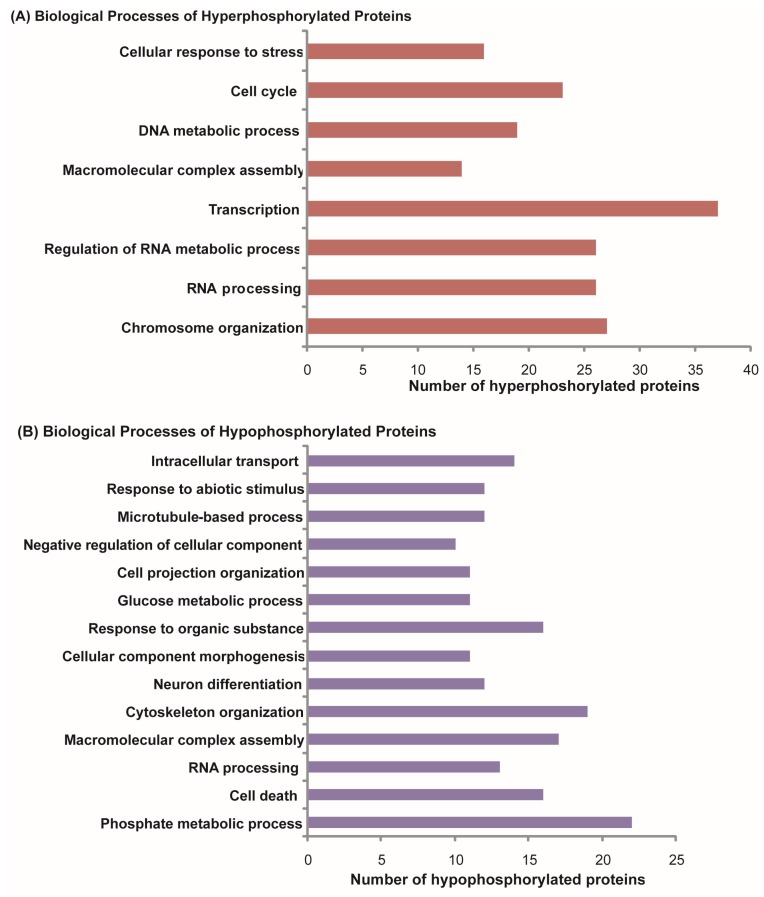
Biological processes enriched in (**A**) hyperphosphorylated proteins and (**B**) hypophosphorylated proteins of RB. Gene ontology (GO) analysis of hyperphosphorylated proteins identified various biological processes required for active cell proliferation such as cell cycle, transcription, and chromosome organization. Biological processes such as cell death and neuron differentiation were found in hypophosphorylated proteins analysis.

Differentially phosphorylated proteins were categorized based on biological processes based on GO annotation to identify the processes activated in retinoblastoma. Hyperphosphorylated proteins were found to be involved in biological processes such as chromosome organization (14%), RNA processing including splicing and transcription (14%), regulation of RNA metabolism (14%), cellcycle (7%), and cellular response to stress (9%) (Figure 2A). Hypophosphorylated proteins were found to be involved in the phosphate metabolic process, cytoskeleton organization, the glucose metabolic process, and cell death (Figure 2B).

### 2.4. Phosphorylated Motifs Identified in Retinoblastoma

The analysis of sequences surrounding phosphorylation sites enabled the identification of phosphorylation motifs for protein kinases. Each kinase scans the target protein sequences for a specific motif and phosphorylates aspecific residue. The identification of phosphorylated motifs is necessary in order to identify the activated upstream kinases, which can then be targeted using kinase inhibitors to treat the disease. 

**Figure 3:**
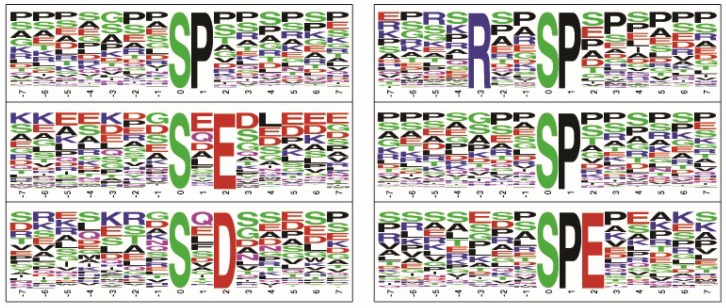
Motif analysis of hyperphosphorylated and hypophosphorylated proteins in RB. Motif analysis identified a proline-directed phosphorylation motif, aspartate and glutamate (acidic aminoacid) directed phosphorylation motifs to be enriched in hyperphosphorylated proteins.

Among the hyperphosphorylated proteins, motifs such as .......SP......, .......S.E....., .......S.D....., ....R..SP...... and .......S.EE.... were identified to be among the top five motifs phosphorylated by kinases (Figure 3). We then carried out NetworKIN analysis to find out the probable upstream kinase of motifs enriched in phosphoproteins identified in retinoblastoma. The probable upstream kinase of the hyperphosphorylated motif “.......SP......” was found to be cyclin-dependent kinases (CDKs). We found CDK1 and CDK11 cyclin-dependent kinases to be hyperphosphorylated in retinoblastoma, indicating their activation. These activated kinases can be targeted using kinase inhibitors for retinoblastoma treatment. Several CDK inhibitors are available for cancer treatment.

### 2.5. Phosphorylated Kinases Identified in Retinoblastoma

**Figure 4:**
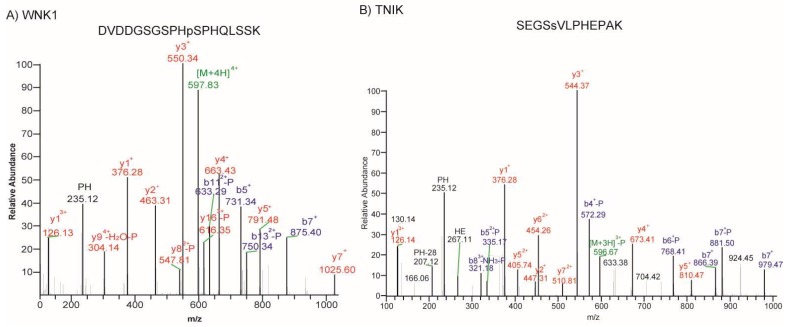
MS/MS spectra of phosphopeptides. Spectral images of hyperphosphorylated kinases. (**A**) WNK1; (**B**) TNIK (TRAF2 and NCK-interacting protein kinase).

Signalling pathways mediated by kinases regulate various hallmark processes in cancer, such as cell cycle progression, angiogenesis, and metastasis. In the past decade, several small-molecule inhibitors have been developed to target key kinases in cancers for therapeutic use. We attempted to identify activated kinases in retinoblastoma, as this would either allow RB researchers to choose already-available kinase inhibitors for the treatment of other cancers or those which can be easily tested in vitro using commercial libraries of kinase inhibitors. Among the differentially regulated phosphoproteins, we identified ten kinases to be hyperphosphorylated, including cyclin-dependant kinases, serine/threonine protein kinase WNK1, TNIK (TRAF2 and NCK-interacting protein kinase), calcium/calmodulin-dependent serine threonine kinase (CASK), discs large MAGUK scaffold protein 3 (DLG3), BRD4, and BAZ1B (Table 2). Several of the identified kinases belong to the class of Ser/Thr kinases and are localized to different compartments of the cell. A Tyr kinase, BAZ1B, was identified that has both nuclear and cytoplasmic localization. Representative spectra of the phosphopeptides corresponding to hyperphosphorylated kinases are presented in Figure 4.

## 3. Discussion

Gene expression studies, copy number variation analysis, and epigenetic profiling including miRNA and methylation of retinoblastoma were carried out to understand the disease mechanism and key players in RB leading to tumour progression. The caveat of these studies is that they do not provide information on activated/inhibited signalling pathways. Hence, we carried out a pilot study on differential phosphoproteomic analysis of retinoblastoma to understand the signalling mechanisms in this cancer. This is the first comprehensive phosphoproteomic analysis of retina and retinoblastoma. Among 1393 proteins identified to be phosphorylated, about 350 proteins were found to be differentially phosphorylated in retinoblastoma compared to normal retina, indicating the retention of a retina-specific signalling signature in addition to tumour-related alterations in cell signalling. 

The insulin signalling pathway was found to be enriched in retina and retinoblastoma tissues. In retina, the insulin pathway is active as apro-survival pathway. Particularly, we identified Akt, FOXO1, and BAD to be phosphorylated in retina and retinoblastoma tissues. Previous studies in retina and neurons identified insulin-regulated Akt to play a major role in cell survival compared to the generally known nutrition storage function of the pathway [25]. It has been established that Akt-mediated phosphorylation of BAD promotes cell survival [26]. We identified the phosphorylation of BAD in retina and retinoblastoma, showing its prominent role in the survival of retina. Targeted studies on retinoblastoma mouse models showed that Rb1 loss and additional oncogenic stress including PTEN loss led to the activation of Akt and the subsequent inactivation of FOXO1, leading to tumour proliferation. On the other hand, in normal retina of mouse models, p-Akt and p-FOXO1 signalling ceased proliferation [27]. Thus, the activation of the insulin signalling pathway had different roles in retina and retinoblastoma.

Functional annotation of hyperphosphorylated proteins found the cellular response to stress to be activated in retinoblastoma. Stress response proteins that were identified to be hyperphosphorylated in retinoblastoma compared to control retina tissues included H2AFX, SIRT1, TNIK, WRNIP1, BAZ1B, and CDK1. H2AFX is a replication-independent histone that contains a histone H2A domain and an SQ-motif. The phosphorylation of H2AFX at Ser140 is denoted as gamma-H2AX and is critical during DNA double strand break signalling. This phosphorylation event helps in the recruitment of repair proteins to carryout repair processes. We identified Ser140 to be hyperphosphorylated in retinoblastoma tissues, indicating the role of H2AFX in signalling DNA damage to recruit repair proteins to the site of damage [28]. We identified the phosphorylation of other proteins which help in sustaining the phosphorylation of H2AFX. For example, HMGA2 protein was found to increase the phosphorylation of H2AFX at serine 140, which is increased by the breakage of double-stranded DNA. We observed the hyperphosphorylation of HMGA2 at Ser101 and Thr150 in retinoblastoma. Similarly, SIRT1, a NAD+ dependent protein deacetylase is required for initial phosphorylation of H2AX [29]. We identified the hyperphosphorylation of SIRT1 at Ser27. The hyperphosphorylation of these proteins is indicative of the activation of the repair system in response to DNA damage usually observed during tumour progression. 

Apart from cellular stress response proteins, cell cycle proteins such as CDK1, CDK11, MCM2, MCM3, MCM5, and CCNB1 were also found to be hyperphosphorylated in retinoblastoma, implicating accelerated cell cycle progression. Besides, we identified DEK protein to be hyperphosphorylated in retinoblastoma. *DEK* is localized in chromosome 6p, the region frequently observed to be amplified in retinoblastoma, which also encompasses another oncogene (*E2F3*) [30]. It has been reported that promoter region of *DEK* has specific E2F binding sites, indicating that *DEK* is transcriptionally activated by E2F. In our study, *DEK* was identified to be hyperphosphorylated at Ser 306 position, the site that lies in the multimerization and DNA binding domain of *DEK* [31]. Draper et al. have shown the role of DEK in regulating apoptosis and senescence in primary human foreskin, HeLa, and osteosarcoma cell line SAOS-2. We can envisage a similar role of regulating apoptosis by DEK in retinoblastoma [32].

Proteins that play a prominent role in the regulation of apoptosis were hypophosphorylated in retinoblastoma compared to retina, indicating a reduction in cell death and favouring tumour cell progression. We observed the hypophosphorylation of proapoptotic proteins including BAD, BCL2L13, NGFR, and ARHGEF6. BCL-2 antagonist of cell death (BAD) belongs to the Bcl-2 family of proteins that regulate cell death machinery. It has been shown in earlier studies that the phosphorylation of BAD at Ser-75 and Ser-118 promotes its binding to 14-3-3 protein. This binding arrests BAD at cytoplasm, leading to the inhibition of BAD-dependent death [33,34,35]. We identified BAD to be hypophosphorylated at Ser118 in retinoblastoma. Phosphoprotein enriched in astrocytes 15 (PEA15) is an antiapoptotic protein which is preferentially phosphorylated at Ser104 and Ser116. PEA15 phosphorylated at Ser116 is reported to bind to FADD (Fas-associated death domain protein), preventing FADD from activating apoptosis [36]. We identified hypophosphorylation at Ser116 of PEA15, which implicates its role in inhibiting apoptosis. It is important to note that the retina is an extension of the central nervous system, and these post-mitotic cells also inhibit apoptosis in order to enable their long-term survival. Thus, proteins relevant to the inhibition of apoptosis were identified to be hypophosphorylated in retinoblastoma compared to control. It has already been well established that post-mitotic cells and cancer cells have parallel survival mechanisms. 

**Figure 5:**
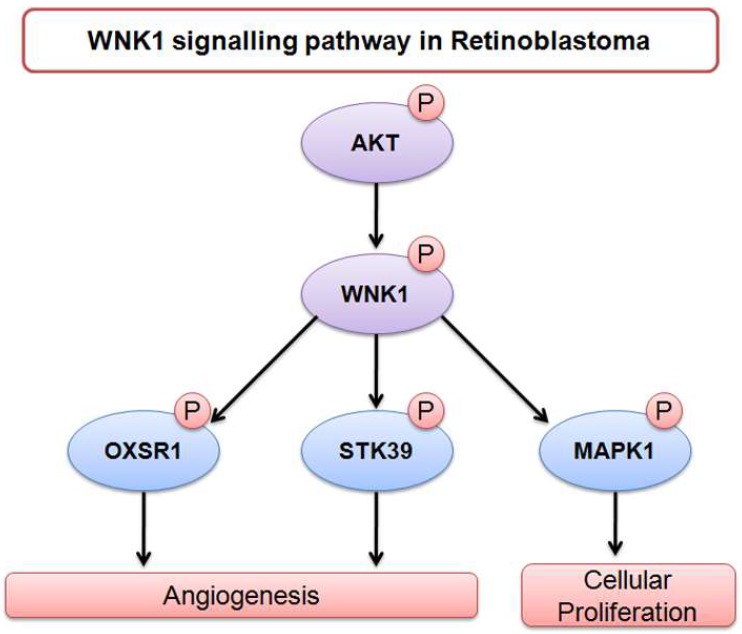
Upstream kinase and downstream targets of WNK1.

More importantly, we identified activated kinases in retinoblastoma for which either preclinical studies or clinical trials are available in other cancers (Table 2). WNK1 is a proposed oncogenic kinase found to be mutated in colorectal adenocarcinoma and breast cancer, whose role in cancer is not fully elucidated [21]. The role of WNK1 in angiogenesis, cell proliferation, and cell migration is recently emerging [37]. WNK1 is the downstream target of VEGFR2 and AK3/PIK pathways, resulting in angiogenesis [38]. The prominent role of WNK1 in proliferation has been shown in mouse neural progenitor cells, where the downregulation of WNK1 resulted in decreased cell proliferation and migration through activation of MAPK, ERK1/2, and/or ERK5 pathways [39]. In our study, WNK1 was identified to be hyperphosphorylated in retinoblastoma. The substrates of WNK1 such as MAPK1, OXSR1, STK39 (SPAK), and ANKS1A were identified to be phosphorylated in our study, but their phosphorylation status was unchanged in retinoblastoma compared to control retina tissues. However, OXSR1 was identified to be overexpressed over two-fold in our proteomics study on retinoblastoma (unpublished data). Dbouk et al. showed in vascular endothelial cells that WNK1-mediated OXSR1 and SPAK activation is essential for proliferation, chemotaxis, and invasion, signifying its role in angiogenesis [40]. Based on the literature evidence, the role of WNK1 and its substrates is depicted in Figure 5. Pertaining to its role in angiogenesis, WNK1 can be a probable therapeutic target for retinoblastoma. 

TNIK belongs to the germinal centre kinase family, and its role in the c-Jun *N*-terminal kinase pathway and the NF-κB pathway is well known [41]. Given its importance in regulating the Wnt pathway, TNIK is considered as a druggable target in colorectal cancer. Small-molecule inhibitors of TNIK have been developed to treat colorectal cancers [42]. Mebendazole, a familiar drug in anti-helminthic ailments, was found to possess anti-cancer properties in pre-clinical models of several cancers, and was shown to inhibit the kinase activity of TNIK [22]. As a result, clinical trials are underway using this drug for the treatment of glioblastoma and paediatric glioma (ClinicalTrials.gov Identifier: NCT01729260 and NCT01837862). Based on these results, TNIK can be considered as a potential therapeutic target for retinoblastoma.

Bromodomain-containing protein 4 (BRD4) is an atypical Ser/Thr protein kinase that belongs to the bromodomain- and extra-terminal domain (BET) family of transcriptional regulators. BRD4 regulates transcription by recruiting nucleosome remodelling complexes and co-activators. BRD4 is known to play an important role in cell cycle progression and has been found to be associated with acetylated chromatin throughout the entire cell cycle. Small-molecule inhibitors of BET protein are now available as treatment for several types of cancer [24]. 

This study serves as preamble for testing those kinase inhibitors in vitro retinoblastoma cell lines and animal models. We believe that these hyperphosphorylated kinases are good targets for therapeutic intervention in retinoblastoma, where targeted therapy has yet not been tried. In addition to these, the present differential phosphoproteomic atlas should enable future molecular diagnostic opportunities such as monitoring gamma-H2AX for disease progression after careful analysis of its role in retinoblastoma.

## 4. Materials and Methods 

### 4.1. Tumour Samples and Lysate Preparation

The present study was approved by the institutional ethics board and conducted at the Medical Research Foundation and Vision Research Foundation, Sankara Nethralaya, India, with Ethics No. 247-2011-P. Control retinas from cadavers were collected from the C.U. SHAH eye bank, Sankara Nethralaya, in the age group of 18–28 years. Tumour tissues were collected with informed consent. The consent and approval to use the samples for research was obtained from the parents/guardians of the patients. The tumour characteristics are given in Supplementary Table S1. In the enucleated eye globe, a part of the tumour was collected for proteomic study, and the other part was used for histopathological studies. The collected tumours were snap frozen in liquid nitrogen and transferred to −80 °C until used for proteomic analyses. Two control retinas and four retinoblastoma tumour tissues were pulverized by adding liquid nitrogen and homogenizing in a homogenizer. The homogenized tissue was lysed by lysis buffers to the final concentration of 2% of SDS in 50 mM Triethyl ammonium bicarbonate (TEABC), phosphatase inhibitors 1 mM sodium fluoride (NaF), 2.5 mM sodium pyrophosphate, 1 mM sodium orthovanadate, and 1 mM β-glycerophosphate. 

### 4.2. Trypsin Digestion and Tandem Mass Tag (TMT) Labelling

Protein estimation was done by bicinchoninic acid (BCA) protein assay kit (Pierce, Waltham, MA, USA) according to the manufacturer’s instructions. Equal amounts of protein (1.5 mg) from normal retina and RB tumours were taken, and sulfhydryl bonds in cysteine were reduced by dithiothreitol (DTT) to the final concentration of 5 mM, and incubated for 20 min at 60 °C. Buffer exchange was performed three times to reduce the concentration of the SDS from 2% to <0.05% by 8M urea buffer in 30 kDa MWCO filters (Millipore, Burlington, MA, USA). Alkylation was carried out with Iodoacetic acid (IAA) to a final concentration of 20 mM for 10 min at room temperature in the dark. The urea in the sample was further removed by buffer exchange with 50 mM TEABC. Digestion of the proteins was performed with sequence-grade trypsin at a 1:20 ratio of trypsin to protein incubated overnight at 37 °C. Peptides were vacuum dried until further use.

For TMT labelling, 0.8 mg of protein was used from each sample. The TMT labels were thawed on ice and reconstituted in 41 µL of anhydrous acetonitrile (ACN). The digested peptide samples were reconstituted in 100 µL of TEABC (pH 8.0). TMT labels 126, 127C, 129C, and 130C were used for labelling RB tumour samples, and TMT labels 129N and 130N were used for labelling control retina samples. The reaction was incubated for 1 h at room temperature. After incubation, the reaction was quenched with 8 µL of 5% hydroxylamine. The labelled peptides were lyophilized and stored until use.

### 4.3. Basic pH Reversed-Phase Liquid Chromatography (bRPLC) and TiO_2_-Based Phosphopeptide Enrichment

Fractionation of the peptides was carried out by bRPLC using XBridge C18, 5 µm, 250 × 4.6 mM column (Waters Corp, Milford, MA, USA). The lyophilized peptides were reconstituted in 2 mL of solvent A (7 mM TEABC pH 9.0). Fractionation was performed by increasing the gradient of solvent B (7 mM TEABC in 90% ACN) on an Agilent 1100 Liquid Chromatography (LC) with a flow rate of 1 mL/min. The fractions were collected in a 96-well plate with 1% formic acid added in the wells. The collected fractions were dried, and to each of the fractions 400 µL of 80% ACN with 0.1% Trifluoroacetic acid (TFA) was used to reconstitute the dried peptides. The fractions were concatenated into 12 fractions and vacuum dried. TiO_2_-based phosphopeptide enrichment was carried out by reconstituting the peptides in 5% DHB solution and mixing with TiO_2_ beads. The peptides and TiO_2_ beads were mixed in a proportion of 1:1 and incubated for 1 h at room temperature. The peptide-bead mixture was centrifuged and saved for further analysis and washed twice with wash solution (3% TFA in 80% ACN). The beads were resuspended in wash solution and transferred onto a stage tip with a C_18_ plug. The beads were washed once with wash solution. The phospho-enriched peptides were eluted twice with 4% ammonium hydroxide into a collection tube containing 4% TFA. The enriched peptides were vacuum dried and stored for downstream analysis.

### 4.4. LC MS/MS Analysis of the Enriched Phosphopeptides

The enriched phosphopeptides were analysed on an LTQ Orbitrap Elite mass spectrometer (Thermo Scientific, Bremen, Germany) interfaced with an Easy nLC II nanoflow LC system. The peptides were reconstituted in 0.1% formic acid and loaded onto the trap column (75 µm × 2 cm) packed in-house with magic C18 AQ. The peptides were resolved using an analytical column (75 µm × 20 cm), and the flow rate was adjusted to 350 nL/min with a linear gradient of 10–35% solvent B (0.1% formic acid and 95% ACN) over 80 minutes. The sample loading and reconditioning of the column was done for 40 minutes, taking the total run time of 120 minutes. Data-dependent acquisition with full scans at 350–1700 *m*/*z* range was carried out using an Orbitrap mass analyser at a mass resolution of 120,000 at 400 *m*/*z*. The fifteen most intense precursor ions were selected for MS/MS fragmentation using high-energy collision-induced dissociation fragmentation (HCD) with 32% normalized collision energy and detected at a mass resolution of 30,000 at 400 *m*/*z*. Dynamic exclusion was set for 30 s with a 10 ppm mass window. Internal calibration was carried out using lock mass option (*m*/*z* 445.1200025) from ambient air. 

### 4.5. Data Analysis 

The Data analysis was performed on Proteome discoverer 1.4(1.4.0.288) (Thermo Fisher Scientific Inc., Bremen, Germany) using Sequest and Mascot search engines. The data was searched against the NCBI Human RefSeq protein database 65 containing71, 644 protein sequences (https://www.ncbi.nlm.nih.gov/refseq/). Fixed modification included in the search parameters were the carbamidomethylation of cysteine, TMT 6-plex labels at the *N*-terminal of peptide (229.16 Da) and lysine side chain (229.16 Da). N-terminal acetylation, oxidation of methionine, phosphorylation at serine, threonine, and tyrosine (+79.966 Da), and were included in the algorithm as variable modification. MS/MS were searched with a precursor mass tolerance of 10 ppm, and the fragment mass tolerance was set to 0.05 Da. The protease used was specified as trypsin, and a maximum of two missed cleavages were allowed. The data was searched against a target decoy database, and the false discovery rate was set to 1% at the peptide level. The TMT ratio for each peptide–spectrum match was calculated by the quantitation node, and the probability of phosphorylation for each Ser/Thr/Tyr site was calculated by the phosphoRS3.1 node in the Proteome Discoverer. Only the phosphopeptides with >75% site localization were considered for the analysis. The peptides with ratios of ≥1.5 in at least three samples were considered as significant differentials and used for further data analysis. MS/MS data associated with the study were submitted to the PRIDE database [43] and are available via ProteomeXchange with identifier PXD007228.

### 4.6. Gene Ontology Analysis

Total phosphoproteins identified in the study were analysed by DAVID to identify the signalling pathways enriched in retina. Pathways with *p*-values <0.05 were considered for discussion. Gene ontology-based functional enrichment analysis was carried out using DAVID. The identified differentially phosphorylated proteins were categorized based on biological process by carrying out bioinformatic analysis using DAVID. Biological processes with at least ten proteins in one cluster for a given GO term and *p*-values <0.05 were considered for discussion. 

### 4.7. Motif Enrichment Analysis

Identified phosphopeptide sequences were formatted to 15-amino-acid sequences with the phosphorylated residue in the central position and seven flanking amino acids. The residues lying in N- and C- termini of the proteins were excluded from the analysis. The remaining peptides were used for motif analysis using the motif-x v1.2 (http://motif-x.med.harvard.edu/) algorithm [44] for cases of serine, threonine, and tryptophan as the central amino acid. All human proteins were chosen as a background database of proteins for significance analysis. Motifs supported by at least ten peptides and *p*-value threshold of 1 × 10^−6^ were considered as enriched in this study. 

### 4.8. Kinase–Substrate Network Analysis

Probable upstream kinases of phosphoproteins with enriched substrate motifs were identified using NetworKIN 3.0 [45]. Offline version NetworKIN was run on a custom input file with protein accessions, phosphorylation positions, and residues. Default parameters were used in NetworKIN analysis and for filtering the results. Thus-identified kinase–substrate interactions were integrated with motif results and differential status of phosphorylation for further analysis and interpretation. 

## Figures and Tables

**Table 1:** Pathways found to be enriched in retina and retinoblastoma (RB).

Pathways	Genes	Count	*p*-Value
Spliceosome	*NCBP1*, *TRA2B*, *PPIL1*, *U2AF2*, *TRA2A*, *HSPA1A*, *SART1*, *SF3B2*, *CTNNBL1*, *HNRNPA3*, *SF3B1*, *DDX46*, *HNRNPK*, *DDX23*, *RBM8A*, *PCBP1*, *USP39*, *DHX16*, *SNRNP70*, *ACIN1*, *HNRNPC*, *PRPF40B*, *RBM25*, *PRPF40A*, *DDX42*, *PRPF31*, *PRPF3*, *SNW1*, *SF3A1*, *HNRNPA1*, *HNRNPU*, *SNRNP200*, *SLU7*, *THOC2*, *PRPF38B*, *RBM17*, *THOC1*, *PRPF38A*	38	4.47 × 10^−14^
Tight junction	*PARD3*, *CASK*, *CLDN11*, *PTEN*, *TJAP1*, *MYL9*, *CTTN*, *MLLT4*, *AKT2*, *PRKCA*, *SYMPK*, *EPB41*, *MPDZ*, *PRKCI*, *MYH9*, *PRKCE*, *CTNNA1*, *CTNNA2*, *PRKCB*, *EPB41L2*, *EPB41L3*, *TJP1*, *EPB41L1*, *MYH14*, *TJP3*, *TJP2*, *SPTAN1*, *MYH10*	28	8.77 × 10^−7^
Insulin signalling pathway	*PHKB*, *FOXO1*, *PPP1R3D*, *PRKAR2B*, *PRKAR2A*, *SORBS1*, *GYS1*, *FASN*, *PRKACA*, *PRKAA1*, *PRKACB*, *AKT2*, *MAP2K1*, *BRAF*, *ACACA*, *PRKCI*, *PRKAB1*, *RAF1*, *BAD*, *MAPK1*, *CRKL*, *PRKAR1B*, *TSC2*, *PRKAR1A*, *MAPK3*, *CRK*, *LIPE*	27	3.44 × 10^−6^
Fc gamma R-mediated phagocytosis	*PRKCA*, *DNM3*, *DNM1L*, *MAP2K1*, *SPHK2*, *LYN*, *MARCKSL1*, *ASAP2*, *ASAP1*, *RAF1*, *PRKCE*, *AMPH*, *PRKCB*, *MAPK1*, *CRKL*, *MAPK3*, *CFL1*, *MARCKS*, *PAK1*, *CRK*, *AKT2*	21	1.20 × 10^−5^
ErbB signalling pathway	*PRKCA*, *EGFR*, *MAP2K1*, *BRAF*, *CAMK2G*, *RAF1*, *BAD*, *PRKCB*, *MAPK1*, *CRKL*, *PAK2*, *PAK4*, *NCK1*, *MAPK3*, *CAMK2D*, *PAK1*, *CRK*, *ABL2*, *AKT2*	19	4.20 × 10^−5^

**Table 2:** List of kinases identified to be hyperphosphorylated in retinoblastoma.

#	Gene Symbol	Kinase	Ser/Thr/Tyr Kinase	Primary Localization	Available Drugs
1	*CDK1*	Cyclin-dependent kinase 1	Ser/Thr protein kinase	Cytoplasm	Flavopiridol, dinaciclib, PD0332991 [18]
2	*CDK11B*	Cyclin-dependent kinase 11B	Ser/Thr protein kinase	Cytoplasm; Nucleus	Proposed target for cancer treatment [19]
3	*WNK1*	Lysine deficient protein kinase 1	Ser/Thr protein kinase	Cytoplasm	Proposed target for cancer treatment [20,21]
4	*TNIK*	TRAF2 and NCK interacting kinase	Ser/Thr protein kinase	Cytoplasm	Mebendazole [22]
5	*BAZ1B*	Bromodomain adjacent to zinc finger domain 1B	Tyrosine kinase	Nucleus; Cytoplasm	Belongs to the bromodomain- and extra terminal domain (BET) family of proteins. Probably targeted by BET inhibitors
6	*PI4K2A*	Phosphatidylinositol 4-kinase type 2 alpha		Golgi apparatus	Small-molecule inhibitors are available for phosphatidylinositol 3-kinase [23]
7	*AAK1*	AP2 associated kinase 1	Ser/Thr protein kinase	Cytoskeleton	-
8	*BRD4*	Bromodomain containing 4	Ser/Thr protein kinase	Nucleus; Cytoplasm	BET inhibitors—JQ1, OTX015, GSK 525762, TEN-010 [24]
9	*CASK*	Calcium/calmodulin-dependent serine threonine kinase	Ser/Thr protein kinase	Plasma membrane	-
10	*DLG3*	Discs large MAGUK scaffold protein 3		Plasma membrane	-

## Supplementary Materials

The following are available online. Table S1: Details of retinoblastoma samples used in the study; Table S2: List of hyperphosphorylated peptides identified in retinoblastoma; Table S3: List of hypophosphorylated peptides identified in retinoblastoma.
