# Supplementary material for: Phosphoproteomics of Retinoblastoma: A Pilot Study Identifies Aberrant Kinases
Source: Molecules. 2018 Jun 15;23(6):1454. doi: 10.3390/molecules23061454 (PMC6100359; doi:10.3390/molecules23061454)
Supplement: Supplementary file 1 [file molecules-23-01454-s001.zip › Supplementary table 1.docx]

**Supplementary table 1:**

Details of RB samples used for the study

| **Sample No.** | **Age (in years)/Sex** | **Laterality** | **Clinico-pathological characteristics** |
| --- | --- | --- | --- |
| RB1 | 3/M | OS | Poorly differentiated, Invasion of Choroid < 3mm, Pre-laminar and laminar. There is no Invasion of post laminar portion. Tumor cells are touching anterior fibres of sclera. Surgical end of optic nerve is free from tumor cells |
| RB2 | 2/M | OD | Poorly differentiated. Tumor seen in the anterior chamber. Tumor cells seen in the iris surface, iris stroma. Tumor seen in angle, trabecular meshwork, ciliary process. Tumor cells seen in Choroid >3mm. There is Pre-Laminar and Laminar invasion of optic nerve by tumor cells. The Post-Laminar and surgical end of optic nerve is free from tumor |
| RB3 | 2/M | OU | Retinoblastoma with Retinoma Formation, Invasion of Choroid Measuring > 3mm. There is Pre-Laminar invasion of optic nerve. The Post- Laminar and Surgical End of Optic Nerve is Free From Tumor Cells |
| RB4 | 0.7/M | OS | Well differentiated Focal Retinal Pigment Epithelial Invasion, Focal Choroidal Invasion Measuring < 3mm. There is Laminar Invasion of Optic Nerve. The Post-Laminar Surgical End of Optic Nerve is free from tumor cells |
